# Introducing Mediterranean Journal of Hematology and Infectious Diseases

**DOI:** 10.4084/MJHID.2009.001

**Published:** 2009-06-17

**Authors:** Giuseppe Leone, Luigi Maria Larocca, Eligio Pizzigallo

**Affiliations:** ^ Istituto di Ematologia; * Istituto di Anatomia Patologica, Università Cattolica del Sacro Cuore, Roma; ° Clinica delle Malattie Infettive, Università di Chieti. Italy

## Abstract

*Mediterranean Journal of Hematology and Infectious Diseases* (MJHID*)* is a new open access, peer-reviewed, online journal, which encompasses different aspects of clinical and translational research providing an insight into the relationship between acute and chronic infections and hematological diseases. MJHID will be a topical journal on subjects of current importance in clinical haematology and infectious diseases. Every issue should have, beside the editor in chief, a guest editor. Both editor in chief and guest editor provide to invite experts in the selected topic to performe a complete update of the arguments readily available for practising phisicians. The journal will have also a section devoted to original papers, case reports and letters to editor and Editorial comment mostly focusing on the arguments treated in the previous topical issues.

Reciprocal interdependence between infectious and haematologic diseases (malignant and non malignant) is well known.^1^,^2^,^3^,^4^ Parassitosis as Malaria, Leishmaniosis, Hookworms and Teniasis infect about a billion people in the word^5^,^6^ and anifest prevalently with anaemia,^7^,^8^ so that they are diagnosed mostly by experienced haematologists on blood or bone marrow smear.^9^ Malaria has been of prominent importance in the diffusion of thalassemia and related disorders in Mediterranean countries and in the rest of the world.^10^ On the other hand, infections are also the main problem in patients affected by hematological malignancies.^11^–^15^ Granulocytopenia, due to bone marrow infiltration or to radio-chemotherapy is the main factor favouring bacterial and fungal infections^16^–^18^ and immunodeficiency, frequent in lymphoproliferative diseases, favours viral, protozoal as well as fungal infections.^19^–^22^ Furthermore, most of the drug utilized to eradicate malignant clones 23–24 have as a side effect immunosuppression, which, on the other hand, is requested to reduce immunological competence in autoimmune blood diseases and to reduce GVH and rejection in Hemopoietic Stem Cell Transplantation.^25^–^27^

Splenectomy frequently performed autoimmune diseases as well in lymphoproliferative diseases is a further cause of immunodeficiency.^28^

Nevertheless, the main cause of immunodeficiency at present is due to HIV infection in developed as well in developing countries.^29^ The blood is the main vector of HIV infection, which become manifest with symptoms related to reduction of T lymphocytes 30–35 and consequent bacterial, viral and fungal and protozoan infections.^36^–^38^ In turn, infections and immunosupression favours malignancies especially of lymphoid tissue in HIV patients^39^–^45^ and, even if in a lower proportion in non HIV.^1^ Worldwide 15 to 20% of cancers are linked to infectious diseases.^1^ In developed Western Countries approximately 10% of all cancers are linked to infectious agents, but they account for as much as 20% of all cancers in developing countries.^1^ It is noteworthy that the first infection driven neoplasia, the bladder cancer, was recognized in egyptian farmers affected by schistosomiasis.^45^ Most of malignancies associated with infections and/or immunosuppression are hematological.^1^ Causative relationship between Epstein-Barr virus infection, virus C hepatitis, HIV, Herpes Virus,^8^ *helicobacter pylori* and B lymphoproliferative diseases are well known^47^–^51^ furthermore association between human T-lymphotropic viruses and adult T-cell leukemias and lymphomas are frequent in in endemic areas.^52^–^53^ Endemic chronic infections are more frequent in developing countries and probably justify the major frequency of cancers linked to infections in these countries.^1^ Although the european and afro-middle oriental mediterranean countries are heterogeneous in life style, income, average life and public health system, and include both developed and developing countries they share epidemiological characteristics and public health problems.^60^ It is evident the interest of Mediterranean European countries to increase the research in the infectious diseases of Mediterranean basin not only for humanitarian reasons but also for their specific interests.^60^ Pathogens can spread far from where they first developed due to increases in trade, international tourism and climate change.^60^–^64^ It is noteworthy that the infections in developing countries are considered a priority in the research program of European Community.^60^ Neglected diseases are a major problem of present medicine. It is now commonly admitted that the so-called (most) neglected tropical diseases have been given little attention. According to World Health Organization, neglected diseases are hidden diseases.^4^–^5^ On a global scale, neglected infectious diseases (NID) are responsible for an estimated 500 000 deaths and millions of disabilities each year. They affect almost exclusively extremely poor populations living in remote areas beyond the reach of health service. Moreover, due to the fact that 90% of cases occur in the low income countries, investment in research on these diseases has been utterly inadequate.^5^ Investing for discover new drugs for these diseases is thought to be not marketable or profitable. However, there is an increasing awareness of this gap, which has been highlighted in a recent report on major and neglected diseases in developing countries prepared by MEP John Bowis and adopted by the European Parliament in 2005.^65^

Urgent pragmatic and efficient measures are needed both at international and national levels. Most of the peoples affected by neglected infectious diseases live in Central Africa, South Asia 5 and, however Mediterranean basin represent from epidemiological point of view an exclusive cross road of Asian, African and European infectious diseases.^60^ Most of neglected infectious diseases are present in Mediterranean basin and their eradication in this area could represent a tremendous incentive to reduce their presence in the rest of the word.

This should be possible bridging the health system gap between the south and the north borders sides of Mediterranean Sea while improving scientific collaboration.

Case report should have educational character. Clinical trials of hematologic and infectious diseases present of Mediterranean area will have their natural home in this Journal. Also case studies relative to important health care intervention in developing countries of south Mediterranean areas will be accepted. All that should contribute to disseminate updated informations among physicians of developing countries on the more frequent diseases of the Mediterranean area.

The results of relevant studies in the field should be made available to scientists and clinicians, in particular those in developing countries where the fight against pathogen related cancers is of the utmost importance.

Every accepted manuscript will be available for reading on its date of acceptance by any person who has access to the web in web-friendly XML. Considering that every issue will be devoted to a topic, when all articles of the same topic will be available they will be formatted and the typeset PDF version will be available. The first issue of the MJHID has been dedicated to “Update in Diagnosis and Treatment of Thalassemia and related Disorders” Guest Editor Prof. Maria Domenica Cappellini.
